# Temporal Generalizability of Machine Learning Models for Predicting Postoperative Delirium Using Electronic Health Record Data: Model Development and Validation Study

**DOI:** 10.2196/50895

**Published:** 2023-10-26

**Authors:** Koutarou Matsumoto, Yasunobu Nohara, Mikako Sakaguchi, Yohei Takayama, Syota Fukushige, Hidehisa Soejima, Naoki Nakashima, Masahiro Kamouchi

**Affiliations:** 1 Biostatistics Center Kurume University Kurume Japan; 2 Big Data Science and Technology Faculty of Advanced Science and Technology Kumamoto University Kumamoto Japan; 3 Department of Nursing Saiseikai Kumamoto Hospital Kumamoto Japan; 4 Department of Inspection Saiseikai Kumamoto Hospital Kumamoto Japan; 5 Institute for Medical Information Research and Analysis Saiseikai Kumamoto Hospital Kumamoto Japan; 6 Medical Information Center Kyushu University Hospital Fukuoka Japan; 7 Department of Health Care Administration and Management Graduate School of Medical Sciences Kyushu University Fukuoka Japan; 8 Center for Cohort Studies Graduate School of Medical Sciences Kyushu University Fukuoka Japan

**Keywords:** postoperative delirium, prediction model, machine learning, temporal generalizability, electronic health record data

## Abstract

**Background:**

Although machine learning models demonstrate significant potential in predicting postoperative delirium, the advantages of their implementation in real-world settings remain unclear and require a comparison with conventional models in practical applications.

**Objective:**

The objective of this study was to validate the temporal generalizability of decision tree ensemble and sparse linear regression models for predicting delirium after surgery compared with that of the traditional logistic regression model.

**Methods:**

The health record data of patients hospitalized at an advanced emergency and critical care medical center in Kumamoto, Japan, were collected electronically. We developed a decision tree ensemble model using extreme gradient boosting (XGBoost) and a sparse linear regression model using least absolute shrinkage and selection operator (LASSO) regression. To evaluate the predictive performance of the model, we used the area under the receiver operating characteristic curve (AUROC) and the Matthews correlation coefficient (MCC) to measure discrimination and the slope and intercept of the regression between predicted and observed probabilities to measure calibration. The Brier score was evaluated as an overall performance metric. We included 11,863 consecutive patients who underwent surgery with general anesthesia between December 2017 and February 2022. The patients were divided into a derivation cohort before the COVID-19 pandemic and a validation cohort during the COVID-19 pandemic. Postoperative delirium was diagnosed according to the confusion assessment method.

**Results:**

A total of 6497 patients (68.5, SD 14.4 years, women n=2627, 40.4%) were included in the derivation cohort, and 5366 patients (67.8, SD 14.6 years, women n=2105, 39.2%) were included in the validation cohort. Regarding discrimination, the XGBoost model (AUROC 0.87-0.90 and MCC 0.34-0.44) did not significantly outperform the LASSO model (AUROC 0.86-0.89 and MCC 0.34-0.41). The logistic regression model (AUROC 0.84-0.88, MCC 0.33-0.40, slope 1.01-1.19, intercept –0.16 to 0.06, and Brier score 0.06-0.07), with 8 predictors (age, intensive care unit, neurosurgery, emergency admission, anesthesia time, BMI, blood loss during surgery, and use of an ambulance) achieved good predictive performance.

**Conclusions:**

The XGBoost model did not significantly outperform the LASSO model in predicting postoperative delirium. Furthermore, a parsimonious logistic model with a few important predictors achieved comparable performance to machine learning models in predicting postoperative delirium.

## Introduction

Delirium occurs in a significant proportion of surgical patients, ranging from 11% to 51% [1]. The risk of postoperative delirium is particularly high among patients who receive general anesthesia [1]. Furthermore, postoperative delirium can lead to a prolonged hospital stay and eventually increase mortality [2,3]. Therefore, it is crucial to accurately estimate the potential risk of postoperative delirium in order to identify high-risk patients prior to surgery.

Various tools have been developed thus far to predict postoperative delirium [4-8]. However, the performance of these risk prediction models is not necessarily sufficient, and it is often difficult to collect the data required for some of the predictors used in these models during routine clinical practice. Moreover, study populations differed depending on the type and timing (planned or emergent) of the surgery. In fact, a previous systematic review and meta-analysis concluded that existing models provide weak evidence and thus are not recommended for clinical practice [5]. Recently, machine learning models have garnered attention in the medical field for their high performance in predicting adverse events [9-32]. Thus, machine learning techniques have been recognized as promising tools for the prediction of postoperative delirium [9-17].

Although data-driven models hold great promise for the future, their implementation in real-world settings remains challenging for several reasons. First, it takes time and effort to monitor outliers and missing values in a large amount of data. Therefore, the implementation of complex machine learning models that rely on hundreds of predictors would be impractical in routine clinical practice. Second, it remains unclear whether complex machine learning models offer any additional value compared to conventional tools in practical applications. Although machine learning models can detect complex interactions and nonlinear relationships between predictors and clinical outcomes, some studies have indicated that these complex machine learning models have limited external validity when compared to traditional logistic regression models [33-35]. The “no free lunch” theorem demonstrates that achieving unbiased models that are highly accurate for all data may not be feasible [36]. Thus, it is still unclear whether a complicated model without interpretability is truly superior to a specific model for a particular problem.

The lack of external validity is a significant barrier to implementing predictive models in clinical practice. Additionally, the accuracy of risk prediction models can deteriorate over time due to covariate shifts. For example, during the COVID-19 pandemic, intensive care unit admission was restricted to isolated patients with COVID-19 and care processes were modified according to the measures to reduce the risk of COVID-19 infection. Consequently, models that were developed to predict postoperative delirium before the COVID-19 pandemic are likely to have had reduced predictive accuracy during the pandemic. Assuming that these factors affect predictive performance, complex machine learning models may also not be useful to predict postoperative delirium in patients hospitalized during the pandemic. Therefore, it would be worth evaluating model performance by temporal validation before and after the COVID-19 pandemic.

Thus, this study aimed to determine (1) whether a complex decision tree ensemble model outperforms a sparse linear regression model in predicting postoperative delirium, and (2) whether machine learning prediction models using copious data are superior to traditional regression models with prespecified predictors. For this purpose, we electronically collected existing data from patients who underwent surgery with general anesthesia at a single hospital in Kumamoto, Japan. We developed machine learning models and a traditional linear regression model using data from a cohort of patients hospitalized before the COVID-19 pandemic and compared their performance in predicting postoperative delirium using data in a cohort of patients hospitalized during the COVID-19 pandemic.

## Methods

### Study Design and Data Sources

We retrospectively reviewed clinical data obtained from patients hospitalized at Saiseikai Kumamoto Hospital, which is designated as an advanced emergency and critical care medical center in Kumamoto City in the southern region of Japan. We accessed, processed, and analyzed clinical data that had been electronically stored within the hospital’s database. We developed the prediction models in accordance with the guidelines outlined in the Transparent Reporting of a Multivariable Prediction Model for Individual Prognosis or Diagnosis statement [37] (Multimedia Appendix 1).

### Ethics Approval

This study was approved by the institutional review board of Saiseikai Kumamoto Hospital (approval number 1072). Owing to the retrospective nature of the study and the use of anonymized clinical data, the requirement for written informed consent was waived. For patients who did not wish to participate in this study, the opportunity to opt out was provided and announced on the Saiseikai Kumamoto Hospital website. During the analysis, patient data were deidentified, and only anonymized information was used. Participation in this study was voluntary, and participants were not compensated for the use of their information.

### Study Patients

**Figure 1:**
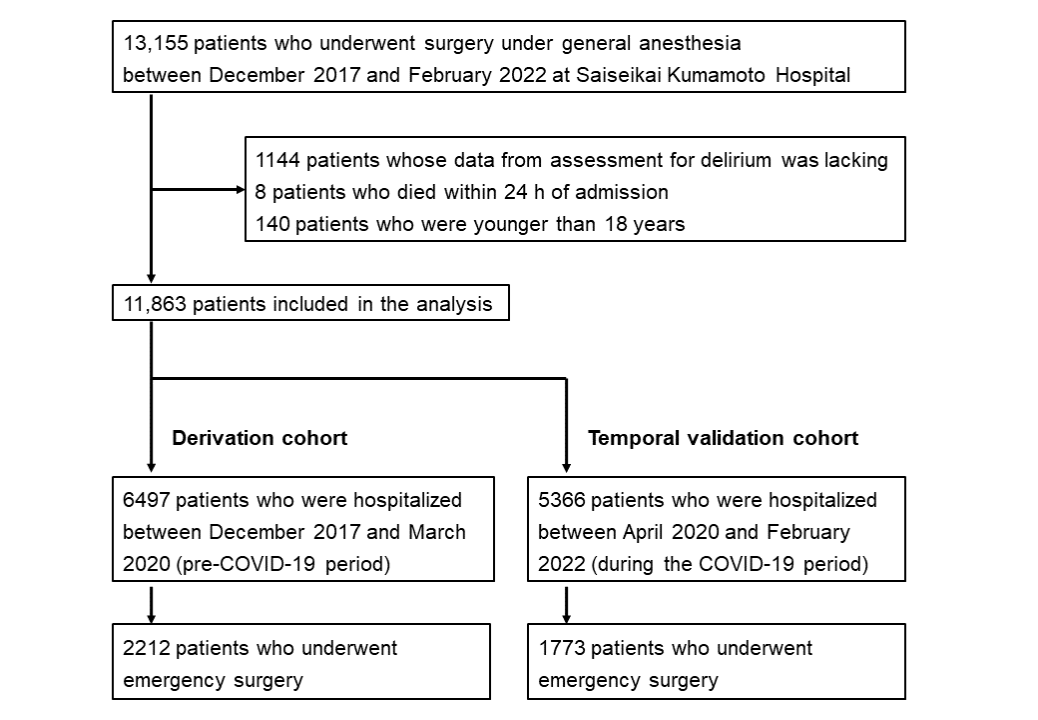
Patient selection flowchart.

We obtained data for a total of 13,155 patients who were admitted to the hospital between December 2017 and February 2022, underwent surgery with general anesthesia, and were discharged before February 2022. Among these patients, we excluded 1144 patients due to missing data on delirium assessment, 8 patients who died within 24 hours of admission, and 140 patients who were younger than 18 years. Finally, a total of 11,863 patients were included in the analysis. A subanalysis was conducted by restricting the patient population to those who underwent emergent surgery (Figure 1).

### Clinical Outcomes

Delirium was diagnosed according to the confusion assessment method (CAM) [38], which is the most widely used instrument for diagnosing delirium [1]. We defined postoperative delirium as the first positive result obtained during hospitalization after surgery using the CAM. Each patient underwent daily assessments using the CAM, starting from the day they were transferred from the operating room to the postoperative care unit. The assessments were conducted by nurses who received training from a delirium assessment committee, which included a psychiatrist.

### Predictors

We collected data on predictors from electronic health record databases, which were routinely accumulated during daily patient care at the hospital. To develop prediction models for patients undergoing surgery under general anesthesia, we used 25 variables that were collected on admission (eg, age, BMI, sex, emergency admission, use of an ambulance, medication, comorbidity, and previous history) as well as during the preoperative (eg, admission ward, catheter, ventilator, physical restraints, and circadian rhythm disorder) and intraoperative (eg, surgery site, anesthesia time, and blood loss during surgery) periods. All variables used for prediction are listed in Multimedia Appendix 2. Additionally, to develop the prediction models for patients who underwent emergent surgery under general anesthesia, we used 30 additional variables, including data derived from blood tests (eg, albumin, creatinine, glucose, hemoglobin, and C-reactive protein levels) and vital sign assessments (eg, systolic blood pressure, pulse rate, SpO_2_, and Glasgow Coma Scale score) upon admission (Multimedia Appendix 2).

### Development of Predictive Models

The extreme gradient boosting (XGBoost) effectively captures nonlinear relationships and interactions between predictors and outcomes [39]. Based on these characteristics, previous studies have reported that XGBoost exhibits a high level of accuracy in predicting postoperative delirium [11-15]. Therefore, we used XGBoost, which is a decision tree ensemble learning method, as a complex machine learning model. Additionally, we also used the least absolute shrinkage and selection operator (LASSO) regression, a sparse linear regression method [40]. LASSO regression serves as a variable selection technique by eliminating regression coefficients of variables that do not significantly contribute to the prediction. To create a machine learning–referenced regression model, we further used logistic regression with prespecified predictors that were identified as the top 10 important variables in both the XGBoost and LASSO models.

The data set contained missing values for several predictors (Multimedia Appendix 2). While multiple imputations are considered the gold standard for handling missing data, its compatibility with decision-tree–based machine learning models is not as straightforward as it is with regression models. Therefore, we used the MissForest method, a type of nonparametric single-imputation approach, for missing value imputation [41]. This method uses other variables, including the outcome, to predict the missing values of the predictor variable using random forest. Since the rate of missing values was at most 8.5%, we used all variables with missing values after imputing them using MissForest. The derivation and the validation cohorts were handled as completely independent data sets, and missing values were imputed within each data set. Additionally, for sensitivity analysis, we conducted a complete case analysis to assess the impact of missing values on the results. Further details regarding the programs used, along with the GitHub URL, are outlined in Multimedia Appendix 3.

### Generalizability Assessment

To verify the general applicability of the prediction model, we performed temporal validation, which is a form of external validation [42,43]. To explore the temporal generalizability, we divided the patients into 2 cohorts based on whether they were admitted to the hospital before or after the onset of the COVID-19 pandemic in Japan. The derivation cohort consisted of patients hospitalized between December 2017 and March 2020, while the validation cohort comprised patients hospitalized between April 2020 and February 2022 (Figure 1).

We developed prediction models using the derivation cohort and then validated the models using the temporal validation cohort. When developing the predictive models in the derivation cohort, the internal validity of the predictive models was assessed using stratified 5-fold cross-validation. The discrimination performance was evaluated based on sensitivity, specificity, positive predictive value, negative predictive value, and area under the receiver operating characteristic curve (AUROC), while the calibration performance was assessed using the calibration slope and intercept of the regression line between the predicted and observed probabilities. The overall performance of the models was measured using the Brier score.

Furthermore, the Matthews correlation coefficient (MCC) and the area under the precision-recall curve (AUPRC) were calculated to provide a fair assessment of binary classification for biased data, such as cases with a small number of events. The MCC takes values between –1 and +1, with a value of +1 indicating perfect agreement between the predicted and observed values, –1 indicating perfect disagreement between the predicted and observed values, and 0 indicating equivalent performance to random prediction [44]. The MCC was defined as follows:

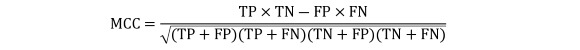



In the equation, TP stands for true positives, TN for true negatives, FP for false positives, and FN for false negatives. For each metric, the 95% CI was calculated using 2000 bootstrap samples.

### Contribution of Predictors to Predictive Performance

We calculated the Shapley additive explanations (SHAP) values to assess the contribution of each predictor in the XGBoost model. SHAP is a model-agnostic machine learning interpretability method that provides a valuable tool for visualizing the contribution of predictors using Shapley values. These values, derived from cooperative game theory, ensure a fair distribution of the contribution across predictors [45]. In the LASSO regression model, we estimated the standardized partial regression coefficients to assess the contribution of each predictor. To evaluate the improvement in predictive performance attributed to each predictor, we compared the AUROC, calibration slope, and calibration intercept after sequentially adding the predictors to the models. The predictive metrics were calculated as each predictor was implemented in the model in the order of its predictive contribution.

### Statistical Analysis

To compare the baseline characteristics, we used the *χ*^2^ test or Fisher exact test for categorical data, the *t* test for data assumed to follow a normal distribution, and the Mann-Whitney *U* test for continuous values when a normal distribution could not be assumed. The Kolmogorov-Smirnov test was used to determine whether the continuous variable data followed the normal distribution. The *t* test was used to evaluate differences in age and BMI between the 2 groups. The 2-sided probability values less than .05 were considered statistically significant. All statistical analyses were conducted using the R statistical package (R Core Team) (Multimedia Appendix 3).

## Results

### Patient Characteristics

The derivation cohort consisted of 6497 patients with a mean age of 68.5 (SD 14.4) years of which 2627 (40.4%) were women. The validation cohort included 5366 patients with a mean age of 67.8 (SD 14.6) years of which 2105 (39.2%) were women. Postoperative delirium occurred in 592 (9.1%) patients in the derivation cohort and in 427 (8%) patients in the validation cohort. Delirium developed within 3 days after surgery in 66% of patients, within a week after surgery in 84% of patients, and within 2 weeks after surgery in 94% of patients.

Table 1 shows the differences in the baseline characteristics between patients with and without postoperative delirium in both the derivation and validation cohorts. In the derivation cohort, all predictors significantly differed, except for central venous port, dialysis catheter, and circadian rhythm disorder. Similar results were observed in the validation cohort, although the frequency of the dialysis catheter differed.

**Table 1:** Differences in the baseline data according to the absence or presence of delirium after surgery.

				Derivation cohort				*P* value		Validation cohort				*P* value
				No delirium (n=5905)		Delirium (n=592)				No delirium (n=4939)		Delirium (n=427)		
**Patient data**														
	Age (years), mean (SD)		67.8 (14.3)		76.0 (12.7)		<.001		67.0 (14.5)		78.0 (12.5)		<.001	
	BMI (kg/m²), mean (SD)		23.2 (3.9)		21.9 (3.8)		<.001		23.3 (4.0)		21.3 (3.8)		<.001	
	Women, n (%)		2358 (39.9)		269 (45.4)		.01		1912 (38.7)		193 (45.2)		.01	
	Emergency admission, n (%)		1814 (30.7)		398 (67.2)		<.001		1474 (29.8)		299 (70.0)		<.001	
	Use of ambulance, n (%)		1110 (18.8)		326 (55.1)		<.001		877 (17.8)		252 (59.0)		<.001	
	**Medication, n (%)**													
		Benzodiazepines	93 (1.7)		18 (3.2)		.02		63 (1.3)		14 (3.3)		.002	
		Opioids	55 (1.0)		29 (5.1)		<.001		40 (0.8)		28 (6.6)		<.001	
		Steroids	62 (1.2)		20 (3.5)		<.001		54 (1.1)		17 (4.0)		<.001	
	Dementia, n (%)		257 (4.8)		88 (15.5)		<.001		176 (3.6)		115 (26.9)		<.001	
	Brain disease, n (%)		509 (9.5)		118 (20.8)		<.001		383 (7.8)		80 (18.7)		<.001	
	**Previous history, n (%)**													
		Heavy drinking	47 (0.9)		12 (2.1)		.009		45 (0.9)		12 (2.8)		.001	
		Delirium	84 (1.6)		32 (5.7)		<.001		94 (1.9)		55 (12.9)		<.001	
**Preoperative data**														
	**Admission ward, n (%)**							<.001						<.001
		General ward (shared room)	2085 (35.3)		123 (20.8)				1666 (33.7)		77 (18.0)			
		General ward (private room)	2940 (49.8)		156 (26.4)				2660 (53.9)		131 (30.7)			
		Intensive care unit	880 (14.9)		313 (52.9)				613 (12.4)		219 (51.3)			
	**Catheter, n (%)**													
		Indwelling urinary catheter	1663 (28.2)		288 (48.6)		<.001		1110 (22.5)		209 (48.9)		<.001	
		Peripheral vein catheter	2856 (48.4)		423 (71.5)		<.001		2136 (43.2)		317 (74.2)		<.001	
		Central venous catheter	322 (5.5)		96 (16.2)		<.001		241 (4.9)		77 (18.0)		<.001	
		Central venous port	39 (0.7)		4 (0.7)		>.99		25 (0.5)		3 (0.7)		.49	
		Dialysis catheter	45 (0.8)		9 (1.5)		.09		36 (0.7)		13 (3.0)		<.001	
		Swan-Ganz catheter	143 (2.4)		47 (7.9)		<.001		81 (1.6)		45 (10.5)		<.001	
	Ventilator, n (%)		4341 (73.5)		500 (84.5)		<.001		3259 (66.0)		351 (82.2)		<.001	
	Physical restraints, n (%)		124 (2.1)		41 (6.9)		<.001		71 (1.4)		40 (9.4)		<.001	
	Circadian rhythm disorder, n (%)		19 (0.3)		2 (0.3)		>.99		27 (0.5)		4 (0.9)		.31	
**Surgical data**														
	**Surgery site, n (%)**							<.001						<.001
		Thoracic cavity and mediastinum	499 (8.5)		7 (1.2)				430 (8.7)		4 (0.9)			
		Chest wall, abdominal wall, and perineum	294 (5.0)		6 (1.0)				264 (5.3)		10 (2.3)			
		Upper abdominal viscera	1421 (24.1)		112 (18.9)				1047 (21.2)		70 (16.4)			
		Lower abdominal viscera	1526 (25.8)		117 (19.8)				1315 (26.6)		107 (25.1)			
		Hip joints and extremities	917 (15.5)		83 (14.0)				1017 (20.6)		78 (18.3)			
		Central nervous system	584 (9.9)		114 (19.3)				358 (7.2)		43 (10.1)			
		Heart and vascular	603 (10.2)		150 (25.3)				480 (9.7)		114 (26.7)			
		Other	61 (1.0)		3 (0.5)				28 (0.6)		1 (0.2)			
	Anesthesia time, min, median (IQR)		209 (137-309)		236 (156-403)		<.001		197 (136-305)		206 (146-379)		<.001	
	Blood loss during surgery (mL), median (IQR)		20 (5-150)		120 (5-703)		<.001		20 (5-100)		70 (10-600)		<.001	

### Predictive Performance of Machine Learning Models

The performance of the prediction models developed using the derivation cohort was first evaluated using stratified 5-fold cross-validation. As a result, the AUROC values for the XGBoost, LASSO, and logistic regression models were found to be 0.85 (SD 0.02), 0.85 (SD 0.02), and 0.854 (SD 0.02), respectively. Thereafter, we validated the performance using the temporal validation cohort of patients hospitalized during the COVID-19 pandemic. The predictive performance of the machine learning models is shown in Table 2. In terms of discrimination performance, the LASSO model showed comparable results to the XGBoost model, with AUROC values ranging 0.86-0.89 and 0.87-0.90, sensitivity of 0.80-0.90 and 0.77-0.91, specificity of 0.73-0.82 and 0.73-0.85, positive predictive value of 0.22-0.28 and 0.22-0.31, and negative predictive value of 0.98-0.99 and 0.98-0.99, respectively. Similarly, MCC values and the AUPRC for the LASSO model ranged from 0.34 to 0.41 and from 0.36 to 0.46, respectively, while the XGBoost model showed values from 0.34 to 0.44 and from 0.36 to 0.46, respectively. Regarding calibration, the LASSO model exhibited good calibration with slope values ranging from 0.98 to 1.14. On the other hand, the XGBoost model displayed a more prominent slope greater than 1, ranging from 1.13 to 1.32, indicating suboptimal calibration of the decision tree ensemble model. Additionally, both the XGBoost and LASSO models demonstrated comparable performance in terms of Brier score, with values ranging from 0.05 to 0.06 for both models.

**Table 2:** Temporal validation of predictive models for delirium after surgery^a^.

		XGBoost^b^	LASSO^c^	LR^d^
**Discriminability metrics**				
	AUROC^e^ (95% CI)	0.88 (0.87 to 0.90)	0.88 (0.86 to 0.89)	0.86 (0.84 to 0.88)
	Sensitivity (95% CI)	0.88 (0.77 to 0.91)	0.84 (0.80 to 0.90)	0.84 (0.75 to 0.87)
	Specificity (95% CI)	0.74 (0.73 to 0.85)	0.78 (0.73 to 0.82)	0.75 (0.74 to 0.84)
	PPV^f^ (95% CI)	0.23 (0.22 to 0.31)	0.25 (0.22 to 0.28)	0.23 (0.22 to 0.29)
	NPV^g^ (95% CI)	0.99 (0.98 to 0.99)	0.98 (0.98 to 0.99)	0.98 (0.97 to 0.99)
**Discriminability metrics for an imbalanced event**				
	MCC^h^ (95% CI)	0.37 (0.34 to 0.44)	0.38 (0.34 to 0.41)	0.35 (0.33 to 0.40)
	AUPRC^i^ (95% CI)	0.41 (0.36 to 0.46)	0.41 (0.36 to 0.46)	0.35 (0.31 to 0.40)
**Calibration metrics**				
	Slope (95% CI)	1.22 (1.13 to 1.32)	1.06 (0.98 to 1.14)	1.10 (1.01 to 1.19)
	Intercept (95% CI)	–0.05 (–0.16 to 0.06)	–0.26 (–0.38 to –0.15)	–0.05 (–0.16 to 0.06)
**Overall metric**				
	Brier score (95% CI)	0.06 (0.05 to 0.06)	0.06 (0.05 to 0.06)	0.06 (0.06 to 0.07)

^a^The predictive models were developed on the training cohorts and validated on the test cohorts. A logistic regression model was developed using the key predictors identified by the machine learning models: age, intensive care unit, neurosurgery, emergency admission, anesthesia time, BMI, blood loss during surgery, and use of an ambulance. The values in parentheses represent 95% CIs after 2000 bootstrap samples.

^b^XGBoost: extreme gradient boosting.

^c^LASSO: least absolute shrinkage and selection operator.

^d^LR: logistic regression.

^e^AUROC: area under the receiver operating characteristic curve.

^f^PPV: positive predictive value.

^g^NPV: negative predictive value.

^h^MCC: Matthews correlation coefficient.

^i^AUPRC: area under the precision-recall curve.

We also validated the predictive performance of the logistic regression model using 8 key predictors—age, intensive care unit, neurosurgery, emergency admission, anesthesia time, BMI, blood loss during surgery, and use of an ambulance—that were identified as important in both the LASSO and XGBoost models (Table 2). The results showed that the logistic regression model also exhibited excellent performance compared with the machine learning models. Discriminability metrics (AUROC 0.84-0.88, sensitivity 0.75-0.87, specificity 0.74-0.84, positive predictive value 0.22-0.29, and negative predictive value 0.97-0.99), discriminability metrics for an imbalanced event (MCC 0.33-0.40 and AUPRC 0.31-0.40), calibration metrics (slope 1.01-1.19 and intercept –0.16 to 0.06), and overall metric (Brier score 0.06-0.07) were comparable to those of the machine learning models.

### Factors Contributing to Prediction

**Figure 2:**
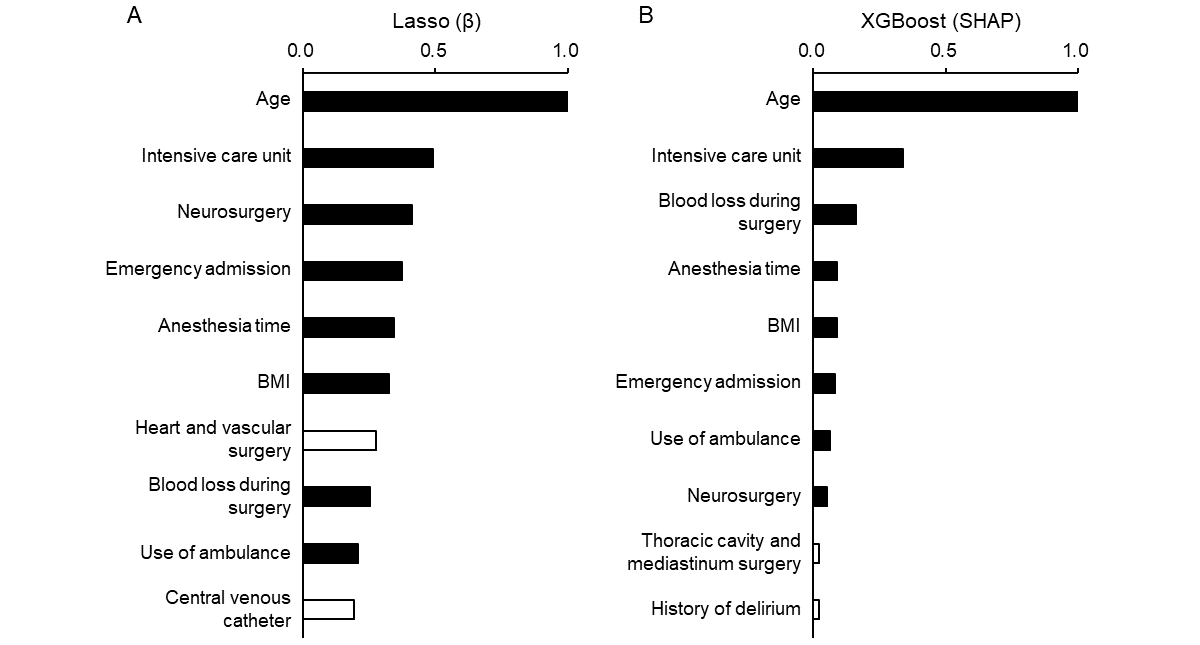
Variable importance for delirium after surgery in the machine learning models. The graph shows the variable importance of the top 10 ranked predictors in the (A) LASSO and (B) XGBoost models. Variable importance was assessed based on the standardized regression coefficient (β) for the LASSO model and the SHAP value for the XGBoost model and is depicted as the value relative to the highest value. Solid bars indicate the variables that are ranked in the top 10 for both models. LASSO: least absolute shrinkage and selection operator; SHAP: Shapley additive explanations; XGBoost: extreme gradient boosting.

Figure 2 illustrates the variable importance of the top 10 predictors identified in both the LASSO and XGBoost models. The ranking of variable importance was similar for each variable in both models. However, some of the high-ranked variables in the LASSO model, such as heart and vascular surgery and central venous catheter, were not necessarily regarded as important in the XGBoost model.

### Predictors and Predictive Performance

**Figure 3:**
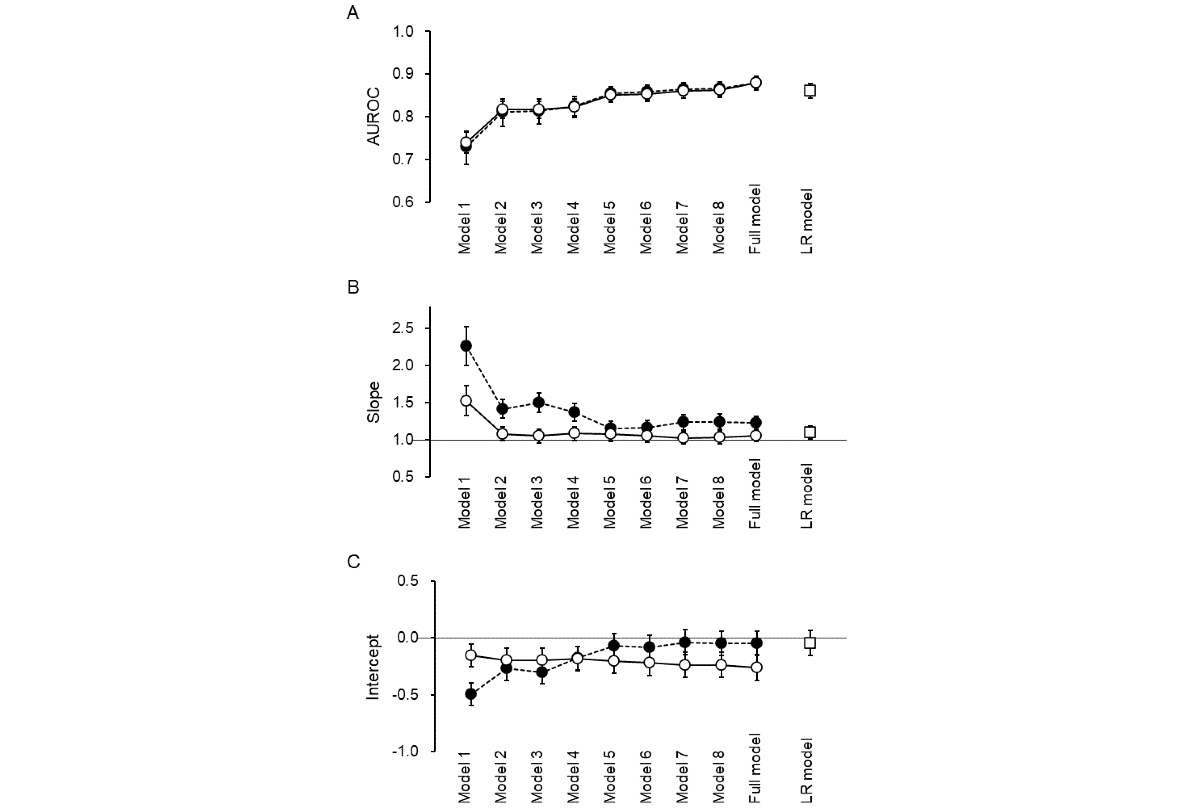
Predictive performance for delirium after surgery of machine learning models and the traditional logistic regression model. The (A) AUROC, (B) calibration slope, and (C) calibration intercept with the increase in the number of predictors are shown for the XGBoost model (closed circles) and LASSO model (open circles) in comparison with those for the logistic regression model using prespecified predictors (open squares). The predictors used in the logistic regression model were those ranked in the top 10 for both the XGBoost and LASSO models. The predictors used in each model are as follows: Model 1: age; Model 2: model 1 + intensive care unit; Model 3: model 2 + neurosurgery; Model 4: model 3 + emergency admission; Model 5: model 4 + anesthesia time; Model 6: model 5 + BMI; Model 7: model 6 + blood loss during surgery; Model 8: model 7 + use of an ambulance; Full model: all 25 variables. AUROC: area under the receiver operating characteristic curve; LASSO: least absolute shrinkage and selection operator; LR: logistic regression; XGBoost: extreme gradient boosting.

Figure 3 demonstrates the impact of including additional predictors on discrimination and calibration. The AUROC did not improve when low-ranked predictors were added to a set of high-ranked predictors in both the LASSO and XGBoost models (Figure 3A).

When calibration is perfect, the slope and the intercept of the calibration plot should be 1 and 0, respectively. The slope in the LASSO model approached 1, whereas the slope in the XGBoost model did not reach 1 even with an increase in the number of predictors (Figure 3B). On the other hand, the intercept in the XGBoost model approached 0 as the number of predictors increased, while it decreased below 0 in the LASSO model (Figure 3C).

### Comparison Between Machine Learning Models and the Logistic Regression Model

We further compared the predictive performance between the machine learning models and the traditional logistic regression model using prespecified predictors. For the logistic regression model, we selected 8 predictors: age, intensive care unit, neurosurgery, emergency admission, anesthesia time, BMI, blood loss during surgery, and use of an ambulance. These predictors were among the top 10 variables associated with postoperative delirium identified by the XGBoost and LASSO models. The logistic regression model, incorporating these 8 predictors, demonstrated sufficient discriminative ability (AUROC 0.84-0.88 and MCC 0.33-0.40), which was comparable to the machine learning models that used all available data (Table 2, Figure 3A). Furthermore, logistic regression with predictors selected based on the machine learning models exhibited better calibration (slope 1.01-1.19 and intercept –0.16 to 0.06) than the XGBoost model (Table 2, Figure 3B). In terms of overall performance (Brier score 0.06-0.07), the logistic regression model was comparable to the machine learning models (Table 2).

### Sensitivity Analysis

We conducted a sensitivity analysis by restricting the study patients to those who underwent emergent surgery (Multimedia Appendix 4). As a result, similar trends were confirmed in these patients. The discriminative ability between the LASSO and XGBoost models was comparable, whereas the XGBoost model showed poor calibration (Multimedia Appendices 5 and 6). Among the top 10 predictors identified by both the XGBoost and LASSO models, 5 predictors (age, intensive care unit, Glasgow Coma Scale score, anesthesia time, and blood loss during surgery) were found in both models (Multimedia Appendix 6). Consequently, the logistic regression model, incorporating these 5 predictors, demonstrated comparable discriminative ability to the machine learning models, while exhibiting better calibration than the XGBoost model (Multimedia Appendices 5 and 6).

Finally, we performed a complete case analysis and found that the results were consistent with the main analysis. The discriminative power among the XGBoost, LASSO, and the logistic regression model was comparable, while the calibration of the XGBoost model was inferior to that of the logistic regression model (Multimedia Appendix 5).

## Discussion

### Principal Results

The temporal generalizability analysis revealed that machine learning exhibited a high discriminative ability in predicting postoperative delirium in a real-world setting. However, increasing the number of predictors did not considerably improve the discriminative performance, even for the machine learning models. The complex ensemble decision tree model did not outperform the sparse linear regression model in terms of discriminative power, and it exhibited poor calibration. In contrast, the traditional logistic regression model with a limited number of important predictors achieved sufficient discriminatory ability in predicting postoperative delirium and demonstrated better calibration than the complex ensemble decision tree model. These findings suggest that a traditional model with prespecified important predictors would be more practical and useful in estimating the risk of postoperative delirium compared to machine learning models.

### Comparison With Prior Work

In this study, we validated the prediction models in terms of temporal generalizability before and after the COVID-19 pandemic, as the health care system underwent significant changes following the pandemic. The pandemic led to a decrease in the number of hospital admissions and treatments for various diseases [46,47].

Additionally, the use of hospital wards, particularly intensive care units, was modified for COVID-19 prevention and control measures. Therefore, we had concerns that the models, especially complex models such as decision tree ensemble models, would overfit the derivation cohort and thus not be applicable to the validation cohort if many predictors were included without a priori verification of the changes in the predictors. Nevertheless, both the decision tree ensemble model and sparse linear regression model exhibited high discriminative power for predicting postoperative delirium. Therefore, machine learning appears to be a useful technique only if we simply categorize patients into high-risk and low-risk groups based on the risk of delirium after surgery using comprehensive data.

In contrast, several studies have shown that machine learning models do not necessarily outperform linear regression models in terms of calibration and generalizability, suggesting that linear regression models may be sufficient in low-dimensional settings with large data sets [33-35]. Furthermore, recent simulation studies on material science data sets have suggested that simple linear regression models are preferable to complex machine learning models, such as a random forest, in terms of extrapolated predictive performance [48]. Our results are consistent with this notion as the linear regression model using only the key predictors accurately predicted postoperative delirium in patients who underwent surgery under general anesthesia. In real-world settings, it would be challenging to manage a large number of predictors, incorporate them into predictive models, and implement prediction systems for postoperative delirium. Instead, it would be convenient if a smaller number of key predictors could provide sufficient accuracy in predicting delirium after surgery. Thus, the implementation of a parsimonious linear regression model rather than a complex machine learning model may be practical and useful in predicting the risk of postoperative delirium.

Although the discriminative ability of the machine learning models and the logistic regression model was comparable, the traditional logistic regression model exhibited better calibration performance than the XGBoost model. In daily clinical practice, poorly calibrated risk estimates can lead to incorrect strategies for safeguarding against postoperative delirium. In this respect, the calibration of the predictive model is crucial for estimating risk for each patient. Flexible models, such as boosted trees, have been reported to be poorly calibrated, and their output prediction probabilities are often corrected using sigmoid functions or isotonic regression [49]. Nevertheless, such a complex conversion process is cumbersome and not cost-effective if prediction systems are implemented in clinical practice.

The implementation of machine learning in real-world settings has gained increasing attention in recent research. Machine learning models have the potential to outperform linear regression models in predicting clinical outcomes when there are nonlinear relationships between the major predictors and the outcomes or when strong interactions exist among the predictors. In such complex scenarios, the predictive accuracy of a flexible model, such as a decision tree ensemble, may surpass that of a linear model. Furthermore, the decision tree ensemble model can effectively handle high-dimensional data without the issues of multicollinearity inherent in linear regression models. Consequently, machine learning techniques offer valuable tools for exploring and identifying important predictors among numerous variables. The selection of appropriate predictive models requires careful consideration of their advantages and disadvantages. Further theoretical and empirical studies are needed to ascertain the use of machine learning models in predicting clinical outcomes in daily clinical practice.

### Limitations

This study had several limitations to be considered. First, some variables contained missing values. Although imputation techniques were used, the results may have been affected—specifically, missing predictor values were imputed using all available data in MissForest. Nevertheless, even when the outcome was excluded in predicting missing values using the imputation method, the results were essentially the same (data not shown). Second, relying on a limited number of predictors obtained during routine clinical work leaves the possibility of unmeasured predictors influencing the outcomes. Third, the analysis was conducted using data from a single center in Japan, necessitating validation of the findings in other settings. Finally, variations in health policies during the COVID-19 pandemic across countries warranted confirmation of the temporal validity in different contexts.

### Conclusions

In recent years, the application of data-driven models to electronic medical record data for outcome prediction has garnered interest. However, the focus has primarily been on complex state-of-the-art machine learning models. In our study, the decision tree ensemble model demonstrated a comparable discriminatory ability to a sparse linear regression model in predicting postoperative delirium using real-world data. Additionally, a simple traditional logistic regression model using known predictors outperformed the complex ensemble decision tree model in terms of calibration. Therefore, a cautious evaluation of the advantages and disadvantages of data-driven models for postoperative delirium prediction is warranted.

## Appendix

Multimedia Appendix 1 TRIPOD (Transparent Reporting of a Multivariable Prediction Model for Individual Prognosis or Diagnosis) checklist.

Multimedia Appendix 2 Missing values in the baseline data.

Multimedia Appendix 3 Supplemental analysis methods.

Multimedia Appendix 4 Differences in baseline data according to the absence or presence of delirium.

Multimedia Appendix 5 Temporal validation of predictive models for delirium.

Multimedia Appendix 6 Predictive performance of machine learning models for delirium after emergent surgery in comparison with a traditional logistic regression model. Variable importance for delirium after emergent surgery in the machine learning models.

## Abbreviations

AUPRC: area under the precision-recall curve
AUROC: area under the receiver operating characteristic curve
CAM: confusion assessment method
LASSO: least absolute shrinkage and selection operator
MCC: Matthews correlation coefficient
MHLW: Ministry of Health, Labour and Welfare
SHAP: Shapley additive explanations
XGBoost: extreme gradient boosting

## Data Availability

The data sets generated and analyzed during this study are available from the corresponding author upon reasonable request. However, owing to the confidential nature of the data, only qualified researchers who are trained in human subject confidentiality protocols may submit requests to use the data set to the corresponding author.
