# Supplementary material for: Temporal Generalizability of Machine Learning Models for Predicting Postoperative Delirium Using Electronic Health Record Data: Model Development and Validation Study
Source: JMIR Perioper Med. 2023 Oct 26;6:e50895. doi: 10.2196/50895 (PMC10636625; doi:10.2196/50895)
Supplement: Multimedia Appendix 2 [file periop_v6i1e50895_app2.docx]

**Table S1**. Missing values in the baseline data for delirium after surgery.

|  | Derivation cohort | |  | Validation cohort | |
| --- | --- | --- | --- | --- | --- |
|  | n = 6497 | Missing |  | n = 5366 | Missing |
| Patient data |  |  |  |  |  |
| Age, y | 68.5 (14.4) | 0.0 |  | 67.8 (14.6) | 0.0 |
| Body mass index, kg/m² | 23.1 (3.9) | 0.6 |  | 23.1 (4.0) | 0.8 |
| Women | 2627 (40.4) | 0.0 |  | 2105 (39.2) | 0.0 |
| Emergency admission | 2212 (34.0) | 0.0 |  | 1773 (33.0) | 0.0 |
| Use of ambulance | 1436 (22.1) | 0.0 |  | 1129 (21.0) | 0.0 |
| Medication |  |  |  |  |  |
| Benzodiazepines | 111 (1.9) | 8.5 |  | 77 (1.4) | 0.1 |
| Opioids | 84 (1.4) | 8.5 |  | 68 (1.3) | 0.1 |
| Steroids | 82 (1.4) | 8.5 |  | 71 (1.3) | 0.1 |
| Dementia | 345 (5.8) | 8.5 |  | 291 (5.4) | 0.1 |
| Brain disease | 627 (10.5) | 8.5 |  | 463 (8.6) | 0.1 |
| Previous history |  |  |  |  |  |
| Heavy drinking | 59 (1.0) | 8.5 |  | 57 (1.1) | 0.1 |
| Delirium | 116 (2.0) | 8.5 |  | 149 (2.8) | 0.1 |
| Preoperative data |  |  |  |  |  |
| Admission ward |  | 0.0 |  |  | 0.0 |
| General ward (shared room) | 2208 (34.0) |  |  | 1743 (32.5) |  |
| General ward (private room) | 3096 (47.7) |  |  | 2791 (52.0) |  |
| Intensive care unit | 1193 (18.4) |  |  | 832 (15.5) |  |
| Catheter |  |  |  |  |  |
| Indwelling urinary catheter | 1951 (30.0) | 0.0 |  | 1319 (24.6) | 0.0 |
| Peripheral vein catheter | 3279 (50.5) | 0.0 |  | 2453 (45.7) | 0.0 |
| Central venous catheter | 418 (6.4) | 0.0 |  | 318 (5.9) | 0.0 |
| Central venous port | 43 (0.7) |  |  | 28 (0.5) |  |
| Dialysis catheter | 54 (0.8) | 0.0 |  | 49 (0.9) | 0.0 |
| Swan-Ganz catheter | 190 (2.9) | 0.0 |  | 126 (2.3) | 0.0 |
| Ventilator | 4841 (74.5) | 0.0 |  | 3610 (67.3) | 0.0 |
| Physical restraints | 165 (2.5) | 0.0 |  | 111 (2.1) | 0.0 |
| Circadian rhythm disorder | 21 (0.3) | 0.0 |  | 31 (0.6) | 0.0 |
| Surgical data |  |  |  |  |  |
| Surgery site |  | 0.0 |  |  | 0.0 |
| Thoracic cavity and mediastinum | 506 (7.8) |  |  | 434 (8.1) |  |
| Chest wall, abdominal wall, perineum | 300 (4.6) |  |  | 274 (5.1) |  |
| Upper abdominal viscera | 1533 (23.6) |  |  | 1117 (20.8) |  |
| Lower abdominal viscera | 1643 (25.3) |  |  | 1422 (26.5) |  |
| Hip joints and extremities | 1000 (15.4) |  |  | 1095 (20.4) |  |
| Central nervous system | 698 (10.7) |  |  | 401 (7.5) |  |
| Heart and vascular | 753 (11.6) |  |  | 594 (11.1) |  |
| Other | 64 (1.0) |  |  | 29 (0.5) |  |
| Anesthesia time, min | 212 (139, 315) | 0.0 |  | 198 (137, 309) | 0.0 |
| Blood loss during surgery, mL | 30 (5, 180) | 0.3 |  | 20 (5, 114) | 0.4 |

Data are shown as mean (SD), n (%), or median (interquartile range). The frequency of missing values is shown as a percentage.

**Table S2**. Missing values in the baseline data for delirium after emergent surgery.

|  | Derivation cohort | |  | Validation cohort | |
| --- | --- | --- | --- | --- | --- |
|  | n = 2212 | Missing |  | n = 1773 | Missing |
| Patient data |  |  |  |  |  |
| Age, y | 68.8 (17.6) | 0.0 |  | 68.5 (17.5) | 0.0 |
| Body mass index, kg/m² | 22.5 (4.17) | 1.6 |  | 22.4 (4.3) | 2.3 |
| Women | 1041 (47.1) | 0.0 |  | 803 (45.3) | 0.0 |
| Use of ambulance | 1433 (64.8) | 0.0 |  | 1128 (63.6) | 0.0 |
| Medication |  |  |  |  |  |
| Benzodiazepines | 45 (2.2) | 6.3 |  | 27 (1.5) | 0.2 |
| Opioids | 64 (3.1) | 6.3 |  | 42 (2.4) | 0.2 |
| Steroids | 46 (2.2) | 6.3 |  | 39 (2.2) | 0.2 |
| Dementia | 259 (12.5) | 6.3 |  | 209 (11.8) | 0.2 |
| Brain disease | 337 (16.3) | 6.3 |  | 208 (11.8) | 0.2 |
| Previous history |  |  |  |  |  |
| Heavy drinking | 44 (2.1) | 6.3 |  | 35 (2.0) | 0.2 |
| Delirium | 57 (2.7) | 6.3 |  | 88 (5.0) | 0.2 |
| Preoperative data |  |  |  |  |  |
| Admission ward |  | 0.0 |  |  | 0.0 |
| General ward (shared room) | 370 (16.7) |  |  | 1743 (32.5) |  |
| General ward (private room) | 762 (34.4) |  |  | 2791 (52.0) |  |
| Intensive care unit | 1080 (48.8) |  |  | 832 (15.5) |  |
| Catheter |  |  |  |  |  |
| Indwelling urinary catheter | 790 (35.7) | 0.0 |  | 524 (29.6) | 0.0 |
| Peripheral vein catheter | 1692 (76.5) | 0.0 |  | 1336 (75.4) | 0.0 |
| Central venous catheter | 159 (7.2) | 0.0 |  | 136 (7.7) | 0.0 |
| Central venous port | 21 (0.9) |  |  | 17 (1.0) |  |
| Dialysis catheter | 38 (1.7) | 0.0 |  | 32 (1.8) | 0.0 |
| Swan-Ganz catheter | 72 (3.3) | 0.0 |  | 61 (3.4) | 0.0 |
| Ventilator | 1746 (78.9) | 0.0 |  | 1404 (79.2) | 0.0 |
| Physical restraints | 141 (6.4) | 0.0 |  | 98 (5.5) | 0.0 |
| Circadian rhythm disorder | 15 (0.7) | 0.0 |  | 27 (1.5) | 0.0 |
| Surgical data |  |  |  |  |  |
| Surgery site |  | 0.0 |  |  | 0.0 |
| Thoracic cavity and mediastinum | 60 (2.7) |  |  | 57 (3.2) |  |
| Chest wall, abdominal wall, perineum | 53 (2.4) |  |  | 55 (3.1) |  |
| Upper abdominal viscera | 388 (17.5) |  |  | 267 (15.1) |  |
| Lower abdominal viscera | 600 (27.1) |  |  | 537 (30.3) |  |
| Hip joints and extremities | 569 (25.7) |  |  | 538 (30.3) |  |
| Central nervous system | 298 (13.5) |  |  | 149 (8.4) |  |
| Heart and vascular | 224 (10.1) |  |  | 166 (9.4) |  |
| Other | 20 (0.9) |  |  | 4 (0.2) |  |
| Anesthesia time, min | 161 (119, 245) | 0.0 |  | 160 (119, 228) | 0.0 |
| Blood loss during surgery, mL | 30 (5, 190) | 0.1 |  | 20 (5, 100) | 0.4 |

Data are shown as mean (SD), n (%), or median (interquartile range). The frequency of missing values is shown as a percentage.

**Table S3**. Missing values in additional baseline data for delirium after emergent surgery.

|  | Derivation cohort | |  | Validation cohort | |
| --- | --- | --- | --- | --- | --- |
|  | n = 2212 | Missing |  | n = 1773 | Missing |
| Vital data |  |  |  |  |  |
| Systolic blood pressure, mmHg | 133 (116, 153) | 0.6 |  | 134 (116, 154) | 0.0 |
| Diastolic blood pressure, mmHg | 77 (66, 88) | 0.7 |  | 79 (68, 91) | 0.0 |
| Pulse rate, /min | 81 (71, 93) | 2.4 |  | 82 (71, 95) | 0.9 |
| SpO_2_, % | 97 (95, 98) | 0.6 |  | 97 (95, 98) | 0.5 |
| Respiratory rate, /min | 18 (16, 21) | 0.7 |  | 18 (16, 22) | 0.4 |
| Body temperature, ℃ | 36.7 (36.3, 37.2) | 0.1 |  | 36.7 (36.4, 37.2) | 0.0 |
| Glasgow Coma Scale score | 15 (14, 15) | 34.2 |  | 15 (14, 15) | 35.0 |
| Laboratory data |  |  |  |  |  |
| Sodium, mmol/L | 139 (137, 141) | 0.7 |  | 140 (137, 141) | 0.7 |
| Potassium, mmol/L | 3.94 (3.61, 4.30) | 0.7 |  | 3.94 (3.63, 4.26) | 0.7 |
| Chloride, mmol/L | 104 (101, 106) | 0.7 |  | 103 (100, 105) | 0.7 |
| Total protein, g/dL | 6.8 (6.3, 7.2) | 0.9 |  | 6.8 (6.3, 7.3) | 1.1 |
| Albumin, g/dL | 3.8 (3.3, 4.2) | 0.9 |  | 3.8 (3.3, 4.2) | 1.1 |
| Albumin/globulin ratio | 1.30 (1.06, 1.54) | 0.9 |  | 1.26 (1.05, 1.46) | 1.1 |
| Blood urea nitrogen, mg/dL | 17.4 (13.1, 24.3) | 0.6 |  | 17.8 (13.4, 25.2) | 0.7 |
| Creatinine, mg/dL | 0.80 (0.63, 1.08) | 0.6 |  | 0.85 (0.68, 1.16) | 0.7 |
| Aspartate aminotransferase, IU/L | 22 (17, 31) | 0.6 |  | 22 (17, 31) | 0.7 |
| Alanine transaminase, IU/L | 17 (12, 27) | 0.6 |  | 18 (12, 27) | 0.7 |
| Lactate dehydrogenase, IU/L | 208 (174, 262) | 0.6 |  | 209 (177, 255) | 0.7 |
| Total bilirubin, mg/dL | 0.80 (0.60, 1.10) | 0.9 |  | 0.80 (0.60, 1.20) | 0.8 |
| Glucose, mg/dL | 134 (113, 170) | 2.8 |  | 128 (108, 161) | 1.8 |
| White blood cells, 10^3^/μL | 9.7 (7.1, 13.2) | 0.5 |  | 9.7 (7.0, 13.1) | 0.6 |
| Red blood cells, 10^6^/μL | 4.2 (3.8, 4.7) | 0.5 |  | 4.2 (3.7, 4.7) | 0.6 |
| Hemoglobin, g/dL | 12.9 (11.3, 14.4) | 0.5 |  | 12.9 (11.2, 14.4) | 0.6 |
| Hematocrit, % | 38.8 (34.5, 42.8) | 0.5 |  | 38.5 (33.7, 42.4) | 0.6 |
| Mean corpuscular volume, fL | 91.9 (88.0, 95.5) | 0.5 |  | 92.00 (88.50, 95.55) | 0.6 |
| Blood platelet count, 10^3^/μL | 20 (16, 25) | 0.5 |  | 22 (18, 27) | 0.6 |
| C-reactive protein, mg/dL | 0.54 (0.08, 4.57) | 1.8 |  | 0.62 (0.09, 5.18) | 2.6 |
| Activated partial thromboplastin time, s | 29.3 (25.6, 34.9) | 2.4 |  | 28.95 (25.80, 34.30) | 2.1 |
| Prothrombin time, s | 12.1 (11.2, 13.2) | 2.4 |  | 12.9 (12.1, 14.3) | 1.9 |
| Prothrombin time-international normalized ratio | 1.01 (0.94, 1.11) | 2.4 |  | 1.01 (0.94, 1.12) | 1.9 |

Data are shown as median (interquartile range). The frequency of missing values is shown as a percentage.
