# Supplementary material for: Temporal Generalizability of Machine Learning Models for Predicting Postoperative Delirium Using Electronic Health Record Data: Model Development and Validation Study
Source: JMIR Perioper Med. 2023 Oct 26;6:e50895. doi: 10.2196/50895 (PMC10636625; doi:10.2196/50895)
Supplement: Multimedia Appendix 3 [file periop_v6i1e50895_app3.docx]

Further details regarding the programs utilized are available on GitHub (https://github.com/kkmmmmm/derilium)

***R Programs for Machine Learning Model***

The data were analyzed using RStudio (http://www.rstudio.com/, version 2022.02.3) with the R statistical package (http://www.r-project.org/, version 4.2.0). For the least absolute shrinkage and selection operator (LASSO) regression, we used the R program “glmnet (https://cran.r-project.org/web/packages/glmnet/glmnet.pdf)” and tuned parameter λ using 10-fold cross-validation with a grid search. The input variables were standardized prior to constructing the likelihood function for the LASSO model. We used the R package “xgboost (https://cran.r-project.org/web/packages/xgboost/xgboost.pdf)” to perform eXtreme Gradient Boosting (XGBoost). In XGBoost, we tuned “nrounds” using 10-fold cross-validation and stopped the training with no improvement in the area under the receiver operating characteristic curve (AUROC) in the test data after five rounds in a row. The parameter “max_depth” was tuned between 1 and 10 with a grid search, and the other parameters were set as follows: eta: 0.1, gamma: 0, colsample_bytree: 1, min_child_weight: 1, and subsample: 1.

***R Programs for Prediction Metrics***

The R package "CalibrationCurves" (https://cran.r-project.org/web/packages/CalibrationCurves/CalibrationCurves.pdf) was used to calculate the AUROC, calibration slope, and calibration intercept. The "PRROC" package (https://cran.r-project.org/web/packages/PRROC/PRROC.pdf) was used to calculate the AUPRC. The "pROC" package (https://cran.r-project.org/web/packages/pROC/pROC.pdf) was used to calculate the number of true positives, true negatives, false positives, and false negatives. The Matthews correlation coefficient (MCC) was then calculated based on these values. The Brier score was calculated using the following formula: $\frac{1}{N}\sum_{i=1}^{N} \left( p_{i}-a_{i} \right)^{2}$, where $p_{i}$ represents the predicted probability of occurrence of an event ranging from 0 to 1, $a_{i}$ denotes the binary outcomes of the event (1 for observed or 0 for not observed), and $N$ is the sample size.

***R Programs for Shapley Additive Explanations (SHAP)***

The R package “shapviz (https://cran.r-project.org/web/packages/shapviz/shapviz.pdf)” was used to calculate and visualize the SHAP values.
