# Supplementary material for: Temporal Generalizability of Machine Learning Models for Predicting Postoperative Delirium Using Electronic Health Record Data: Model Development and Validation Study
Source: JMIR Perioper Med. 2023 Oct 26;6:e50895. doi: 10.2196/50895 (PMC10636625; doi:10.2196/50895)
Supplement: Multimedia Appendix 4 [file periop_v6i1e50895_app4.docx]

**Table S4**. Differences in baseline data according to the absence or presence of delirium after emergent surgery.

|  | Derivation cohort | |  |  | Validation cohort | |  |
| --- | --- | --- | --- | --- | --- | --- | --- |
|  | No delirium | Delirium |  |  | No delirium | Delirium |  |
|  | n = 1814 | n = 398 | P value |  | n = 1474 | n = 299 | P value |
| Patient data |  |  |  |  |  |  |  |
| Age, y | 67.2 (18.0) | 76.2 (13.6) | <0.001 |  | 66.4 (17.5) | 78.8 (13.4) | <0.001 |
| Body mass index, kg/m² | 22.6 (4.2) | 21.8 (3.9) | <0.001 |  | 22.7 (4.4) | 21.0 (3.9) | <0.001 |
| Women | 839 (46.3) | 202 (50.8) | 0.115 |  | 655 (44.4) | 148 (49.5) | 0.124 |
| Use of ambulance | 1107 (61.0) | 326 (81.9) | <0.001 |  | 876 (59.4) | 252 (84.3) | <0.001 |
| Medication |  |  |  |  |  |  |  |
| Benzodiazepines | 36 (2.1) | 9 (2.4) | 0.915 |  | 23 (1.6) | 19 (6.4) | <0.001 |
| Opioids | 41 (2.4) | 23 (6.1) | <0.001 |  | 28 (1.9) | 11 (3.7) | 0.091 |
| Steroids | 32 (1.9) | 14 (3.7) | 0.050 |  | 113 (7.7) | 96 (32.1) | <0.001 |
| Dementia | 182 (10.7) | 77 (20.3) | <0.001 |  | 147 (10.0) | 61 (20.4) | <0.001 |
| Brain disease | 241 (14.2) | 96 (25.3) | <0.001 |  | 23 (1.6) | 19 (6.4) | <0.001 |
| Previous history |  |  |  |  |  |  |  |
| Heavy drinking | 32 (1.9) | 12 (3.2) | 0.173 |  | 27 (1.8) | 8 (2.7) | 0.469 |
| Delirium | 38 (2.2) | 19 (5.0) | 0.005 |  | 50 (3.4) | 38 (12.7) | <0.001 |
| Preoperative data |  |  |  |  |  |  |  |
| Admission ward |  |  | <0.001 |  |  |  | <0.001 |
| General ward (shared room) | 343 (18.9) | 27 (6.8) |  |  | 171 (11.6) | 14 (4.7) |  |
| General ward (private room) | 703 (38.8) | 59 (14.8) |  |  | 764 (51.8) | 67 (22.4) |  |
| Intensive care unit | 768 (42.3) | 312 (78.4) |  |  | 539 (36.6) | 218 (72.9) |  |
| Catheter |  |  |  |  |  |  |  |
| Indwelling urinary catheter | 591 (32.6) | 199 (50.0) | <0.001 |  | 370 (25.1) | 154 (51.5) | <0.001 |
| Peripheral vein catheter | 1378 (76.0) | 314 (78.9) | 0.237 |  | 1088 (73.8) | 248 (82.9) | 0.001 |
| Central venous catheter | 105 (5.8) | 54 (13.6) | <0.001 |  | 85 (5.8) | 51 (17.1) | <0.001 |
| Central venous port | 18 (1.0) | 3 (0.8) | 1.000 |  | 16 (1.1) | 1 (0.3) | 0.335 |
| Dialysis catheter | 30 (1.7) | 8 (2.0) | 0.778 |  | 24 (1.6) | 8 (2.7) | 0.316 |
| Swan-Ganz catheter | 46 (2.5) | 26 (6.5) | <0.001 |  | 34 (2.3) | 27 (9.0) | <0.001 |
| Ventilator | 1421 (78.3) | 325 (81.7) | 0.160 |  | 1154 (78.3) | 250 (83.6) | 0.047 |
| Physical restraints | 102 (5.6) | 39 (9.8) | 0.003 |  | 61 (4.1) | 37 (12.4) | <0.001 |
| Circadian rhythm disorder | 14 (0.8) | 1 (0.3) | 0.496 |  | 25 (1.7) | 2 (0.7) | 0.297 |
| Surgical data |  |  |  |  |  |  |  |
| Surgery site |  |  | <0.001 |  |  |  | <0.001 |
| Thoracic cavity and mediastinum | 58 (3.2) | 2 (0.5) |  |  | 56 (3.8) | 1 (0.3) |  |
| Chest wall, abdominal wall, perineum | 50 (2.8) | 3 (0.8) |  |  | 52 (3.5) | 3 (1.0) |  |
| Upper abdominal viscera | 321 (17.7) | 67 (16.8) |  |  | 219 (14.9) | 48 (16.1) |  |
| Lower abdominal viscera | 509 (28.1) | 91 (22.9) |  |  | 446 (30.3) | 91 (30.4) |  |
| Hip joints and extremities | 505 (27.8) | 64 (16.1) |  |  | 472 (32.0) | 66 (22.1) |  |
| Central nervous system | 212 (11.7) | 86 (21.6) |  |  | 116 (7.9) | 33 (11.0) |  |
| Heart and vascular | 142 (7.8) | 82 (20.6) |  |  | 109 (7.4) | 57 (19.1) |  |
| Other | 17 (0.9) | 3 (0.8) |  |  | 4 (0.3) | 0 (0.0) |  |
| Anesthesia time, min | 154 (115, 228) | 203 (144, 339) | <0.001 |  | 156 (116, 218) | 175 (136, 261) | <0.001 |
| Blood loss during surgery, mL | 20 (5, 150) | 100 (5, 590) | <0.001 |  | 10 (5, 100) | 50 (8, 300) | <0.001 |

Data are shown as mean (SD), n (%), or median (interquartile range).

**Table S5**. Differences in additional baseline data according to the absence or presence of delirium after emergent surgery.

|  | Derivation cohort | |  |  | Validation cohort | |  |
| --- | --- | --- | --- | --- | --- | --- | --- |
|  | No delirium | Delirium |  |  | No delirium | Delirium |  |
|  | n = 1814 | n = 398 | P value |  | n = 1474 | n = 299 | P value |
| Vital data |  |  |  |  |  |  |  |
| Systolic blood pressure, mmHg | 135 (117, 153) | 129 (112, 150) | 0.002 |  | 136 (119, 155) | 128 (107, 150) | <0.001 |
| Diastolic blood pressure, mmHg | 77 (66, 89) | 75 (64, 87) | 0.019 |  | 80 (70, 91) | 74 (63, 90) | <0.001 |
| Pulse rate, /min | 80 (70, 93) | 86 (72, 97) | 0.002 |  | 81 (71, 95) | 85 (74, 99) | 0.004 |
| SpO_2_, % | 97 (96, 98) | 97 (95, 99) | 0.017 |  | 97 (96, 98) | 96 (94, 98) | <0.001 |
| Respiratory rate, /min | 18 (16, 20) | 19 (16, 23) | <0.001 |  | 18 (16, 21) | 20 (16, 25) | <0.001 |
| Body temperature, ℃ | 36.8 (36.4, 37.2) | 36.5 (36.1, 37.0) | <0.001 |  | 36.7 (36.4, 37.2) | 36.6 (36.2, 37.1) | 0.01 |
| Glasgow Coma Scale score | 15 (15, 15) | 15 (12, 15) | <0.001 |  | 15 (15, 15) | 14 (13, 15) | <0.001 |
| Laboratory data |  |  |  |  |  |  |  |
| Sodium, mmol/L | 139 (137, 141) | 139 (137, 141) | 0.356 |  | 140 (137, 141) | 139 (136, 141) | 0.071 |
| Potassium, mmol/L | 3.96 (3.64, 4.30) | 3.84 (3.50, 4.29) | 0.002 |  | 3.93 (3.64, 4.24) | 3.98 (3.53, 4.42) | 0.543 |
| Chloride, mmol/L | 104 (101, 106) | 104 (101, 106) | 0.334 |  | 103 (100, 105) | 102 (99, 105) | 0.038 |
| Total protein, g/dL | 6.8 (6.4, 7.3) | 6.6 (6.0, 7.1) | <0.001 |  | 6.9 (6.5, 7.3) | 6.5 (5.9, 7.0) | <0.001 |
| Albumin, g/dL | 3.9 (3.4, 4.3) | 3.6 (3.1, 4.1) | <0.001 |  | 3.9 (3.4, 4.2) | 3.4 (2.9, 3.8) | <0.001 |
| Albumin/globulin ratio | 1.31 (1.07, 1.56) | 1.25 (1.03, 1.46) | <0.001 |  | 1.28 (1.06, 1.48) | 1.13 (0.94, 1.33) | <0.001 |
| Blood urea nitrogen, mg/dL | 16.9 (12.8, 23.4) | 19.4 (14.8, 26.6) | <0.001 |  | 17.1 (13.1, 23.4) | 23.7 (16.9, 34.4) | <0.001 |
| Creatinine, mg/dL | 0.79 (0.62, 1.03) | 0.86 (0.66, 1.17) | 0.005 |  | 0.83 (0.67, 1.09) | 1.01 (0.75, 1.51) | <0.001 |
| Aspartate aminotransferase, IU/L | 22 (17, 31) | 23 (18, 34) | 0.002 |  | 22 (17, 30) | 23 (18, 34) | 0.007 |
| Alanine transaminase, IU/L | 18 (12, 27) | 17 (12, 25) | 0.109 |  | 18 (13, 28) | 16 (11, 25) | 0.002 |
| Lactate dehydrogenase, IU/L | 206 (171, 258) | 222 (188, 278) | <0.001 |  | 206 (173, 248) | 231 (199, 284) | <0.001 |
| Total bilirubin, mg/dL | 0.80 (0.60, 1.10) | 0.80 (0.60, 1.00) | 0.893 |  | 0.80 (0.60, 1.20) | 0.80 (0.60, 1.20) | 0.732 |
| Glucose, mg/dL | 132 (112, 163) | 151 (121, 191) | <0.001 |  | 126 (107, 158) | 144 (113, 178) | <0.001 |
| White blood cells, 10^3^/μL | 9.8 (7.1, 13.3) | 9.7 (7.1, 12.9) | 0.494 |  | 9.7 (7.0, 13.0) | 9.8 (6.8, 13.1) | 0.638 |
| Red blood cells, 10^6^/μL | 4.3 (3.8, 4.7) | 4.1 (3.6, 4.6) | <0.001 |  | 4.3 (3.7, 4.7) | 3.9 (3.4, 4.4) | <0.001 |
| Hemoglobin, g/dL | 13.0 (11.4, 14.5) | 12.5 (11.1, 14.0) | 0.001 |  | 13.2 (11.4, 14.6) | 12.0 (10.5, 13.4) | <0.001 |
| Hematocrit, % | 39.0 (34.7, 43.0) | 38.2 (33.9, 41.7) | 0.001 |  | 39.1 (34.4, 42.9) | 35.8 (31.7, 40.4) | <0.001 |
| Mean corpuscular volume, fL | 91.6 (87.8, 95.4) | 92.9 (90.0, 96.1) | <0.001 |  | 91.9 (88.4, 95.3) | 92.7 (89.6, 96.5) | 0.007 |
| Blood platelet count, 10^3^/μL | 21 (17, 25) | 19 (15, 23) | <0.001 |  | 22 (18, 27) | 20 (15, 25) | <0.001 |
| C-reactive protein, mg/dL | 0.53 (0.08, 4.25) | 0.58 (0.09, 5.50) | 0.594 |  | 0.57 (0.09, 4.89) | 1.23 (0.11, 7.54) | 0.012 |
| Activated partial thromboplastin time, s | 29.3 (25.6, 34.7) | 29.5 (25.3, 36.4) | 0.574 |  | 28.70 (25.70, 33.65) | 30.70 (26.60, 37.30) | <0.001 |
| Prothrombin time, s | 12.0 (11.2, 13.1) | 12.3 (11.4, 13.8) | <0.001 |  | 12.8 (12.0, 14.0) | 13.7 (12.7, 15.8) | <0.001 |
| Prothrombin time-international normalized ratio | 1.01 (0.94, 1.10) | 1.04 (0.95, 1.16) | <0.001 |  | 1.00 (0.93, 1.10) | 1.07 (0.98, 1.23) | <0.001 |

Data are shown as median (interquartile range).
