# Supplementary material for: Temporal Generalizability of Machine Learning Models for Predicting Postoperative Delirium Using Electronic Health Record Data: Model Development and Validation Study
Source: JMIR Perioper Med. 2023 Oct 26;6:e50895. doi: 10.2196/50895 (PMC10636625; doi:10.2196/50895)
Supplement: Multimedia Appendix 5 [file periop_v6i1e50895_app5.docx]

**Table S6.** Temporal validation of predictive models for delirium after emergent surgery.

|  |  | **XGBoost** | **LASSO** | **LR** |
| --- | --- | --- | --- | --- |
| **Discriminability metrics** | AUROC (95% CI) | 0.81 (0.79, 0.83) | 0.83 (0.80, 0.85) | 0.80 (0.77, 0.82) |
|  | Sensitivity (95% CI) | 0.86 (0.78, 0.91) | 0.85 (0.78, 0.92) | 0.74 (0.65, 0.89) |
|  | Specificity (95% CI) | 0.64 (0.59, 0.71) | 0.66 (0.60, 0.74) | 0.71 (0.55, 0.80) |
|  | PPV (95% CI) | 0.32 (0.30, 0.36) | 0.34 (0.31, 0.39) | 0.34 (0.28, 0.40) |
|  | NPV (95% CI) | 0.96 (0.94, 0.97) | 0.96 (0.94, 0.97) | 0.93 (0.92, 0.96) |
| **Discriminability metrics for an imbalanced event** | MCC (95% CI) | 0.37 (0.34, 0.42) | 0.39 (0.36, 0.44) | 0.35 (0.31, 0.40) |
|  | AUPRC (95% CI) | 0.41 (0.35, 0.46) | 0.48 (0.42, 0.54) | 0.43 (0.37, 0.49) |
| **Calibration metrics** | Slope (95% CI) | 1.47 (1.28, 1.66) | 1.02 (0.89, 1.14) | 0.99 (0.86, 1.12) |
|  | Intercept (95% CI) | -0.14 (-0.27, -0.01) | -0.18 (-0.32, -0.04) | 0.04 (-0.09, 0.18) |
| **Overall metric** | Brier score (95% CI) | 0.12 (0.11, 0.13) | 0.11 (0.10, 0.12) | 0.12 (0.11, 0.13) |

AUROC: area under the receiver operating characteristic curve, PPV: positive predictive value, NPV: negative predictive value, MCC: Matthews correlation coefficient, AUPRC: area under the precision-recall curve, CI: confidence interval, XGBoost: eXtreme Gradient Boosting, LASSO: Least Absolute Shrinkage and Selection Operator regression, LR: logistic regression.

The predictive models were developed using the training cohorts and validated using the test cohorts. The logistic regression model was developed using the key predictors identified by the machine learning models: age, intensive care unit, Glasgow Coma Scale score, anesthesia time, and blood loss during surgery. The values in parentheses represent 95% confidence intervals after 2000 bootstrap samples.

**Table S7.** Temporal validation of predictive models for delirium after surgery in complete case analysis.

|  |  | **XGBoost** | **LASSO** | **LR** |
| --- | --- | --- | --- | --- |
| **Discriminability metrics** | AUROC (95% CI) | 0.88 (0.87, 0.90) | 0.88 (0.86, 0.90) | 0.86 (0.85, 0.88) |
|  | Sensitivity (95% CI) | 0.82 (0.78, 0.89) | 0.82 (0.78, 0.91) | 0.82 (0.75, 0.87) |
|  | Specificity (95% CI) | 0.80 (0.74, 0.83) | 0.79 (0.70, 0.83) | 0.77 (0.74, 0.84) |
|  | PPV (95% CI) | 0.26 (0.22, 0.29) | 0.25 (0.20, 0.28) | 0.24 (0.22, 0.29) |
|  | NPV (95% CI) | 0.98 (0.98, 0.99) | 0.98 (0.98, 0.99) | 0.98 (0.98, 0.99) |
| **Discriminability metrics for an imbalanced event** | MCC (95% CI) | 0.38 (0.34, 0.42) | 0.38 (0.33, 0.41) | 0.36 (0.33, 0.41) |
|  | AUPRC (95% CI) | 0.42 (0.37, 0.46) | 0.41 (0.36, 0.46) | 0.35 (0.30, 0.40) |
| **Calibration metrics** | Slope (95% CI) | 1.19 (1.09, 1.28) | 1.06 (0.97, 1.14) | 1.13 (1.04, 1.22) |
|  | Intercept (95% CI) | -0.05 (-0.16, 0.06) | -0.34 (-0.45, -0.22) | -0.06 (-0.16, 0.06) |
| **Overall metric** | Brier score (95% CI) | 0.06 (0.05, 0.06) | 0.06 (0.05, 0.06) | 0.06 (0.06, 0.07) |

AUROC: area under the receiver operating characteristic curve, PPV: positive predictive value, NPV: negative predictive value, MCC: Matthews correlation coefficient, AUPRC: area under the precision-recall curve, CI: confidence interval, XGBoost: eXtreme Gradient Boosting, LASSO: Least Absolute Shrinkage and Selection Operator regression, LR: logistic regression.

The predictive models were developed using the training cohorts and validated using the test cohorts. The logistic regression model was developed using the key predictors identified by the machine learning models: age, intensive care unit, neurosurgery, emergency admission, anesthesia time, body mass index, blood loss during surgery, and use of an ambulance. The values in parentheses represent 95% confidence intervals after 2,000 bootstrap samples.

**Table S8.** Temporal validation of predictive models for delirium after emergent surgery in complete case analysis.

|  |  | **XGBoost** | **LASSO** | **LR** |
| --- | --- | --- | --- | --- |
| **Discriminability metrics** | AUROC (95% CI) | 0.79 (0.76, 0.82) | 0.81 (0.78, 0.84) | 0.78 (0.75, 0.81) |
|  | Sensitivity (95% CI) | 0.81 (0.77, 0.93) | 0.89 (0.68, 0.94) | 0.75 (0.58, 0.86) |
|  | Specificity (95% CI) | 0.65 (0.52, 0.69) | 0.58 (0.53, 0.81) | 0.68 (0.57, 0.85) |
|  | PPV (95% CI) | 0.38 (0.34, 0.41) | 0.36 (0.34, 0.48) | 0.38 (0.34, 0.51) |
|  | NPV (95% CI) | 0.93 (0.92, 0.97) | 0.95 (0.90, 0.97) | 0.91 (0.88, 0.94) |
| **Discriminability metrics for an imbalanced event** | MCC (95% CI) | 0.38 (0.34, 0.43) | 0.38 (0.35, 0.46) | 0.35 (0.31, 0.44) |
|  | AUPRC (95% CI) | 0.45 (0.38, 0.51) | 0.48 (0.42, 0.55) | 0.45 (0.39, 0.52) |
| **Calibration metrics** | Slope (95% CI) | 1.22 (1.02, 1.43) | 1.10 (0.93, 1.27) | 0.99 (0.82, 1.16) |
|  | Intercept (95% CI) | 0.02 (-0.14, 0.18) | -0.03 (-0.19, 0.14) | 0.09 (-0.07, 0.25) |
| **Overall metric** | Brier score (95% CI) | 0.14 (0.13, 0.15) | 0.13 (0.12, 0.15) | 0.14 (0.13, 0.15) |

AUROC: area under the receiver operating characteristic curve, PPV: positive predictive value, NPV: negative predictive value, MCC: Matthews correlation coefficient, AUPRC: area under the precision-recall curve, CI: confidence interval, XGBoost: eXtreme Gradient Boosting, LASSO: Least Absolute Shrinkage and Selection Operator regression LR: logistic regression.

The predictive models were developed using the training cohorts and validated using the test cohorts. The logistic regression model was developed using the key predictors identified by the machine learning models: age, intensive care unit, Glasgow Coma Scale score, anesthesia time, and blood loss during surgery. The values in parentheses represent 95% confidence intervals after 2000 bootstrap samples.
