# Supplementary material for: Temporal Generalizability of Machine Learning Models for Predicting Postoperative Delirium Using Electronic Health Record Data: Model Development and Validation Study
Source: JMIR Perioper Med. 2023 Oct 26;6:e50895. doi: 10.2196/50895 (PMC10636625; doi:10.2196/50895)
Supplement: Multimedia Appendix 6 [file periop_v6i1e50895_app6.docx]

**Figure S1**. Predictive performance of machine learning models for delirium after emergent surgery in comparison with a traditional logistic regression model.


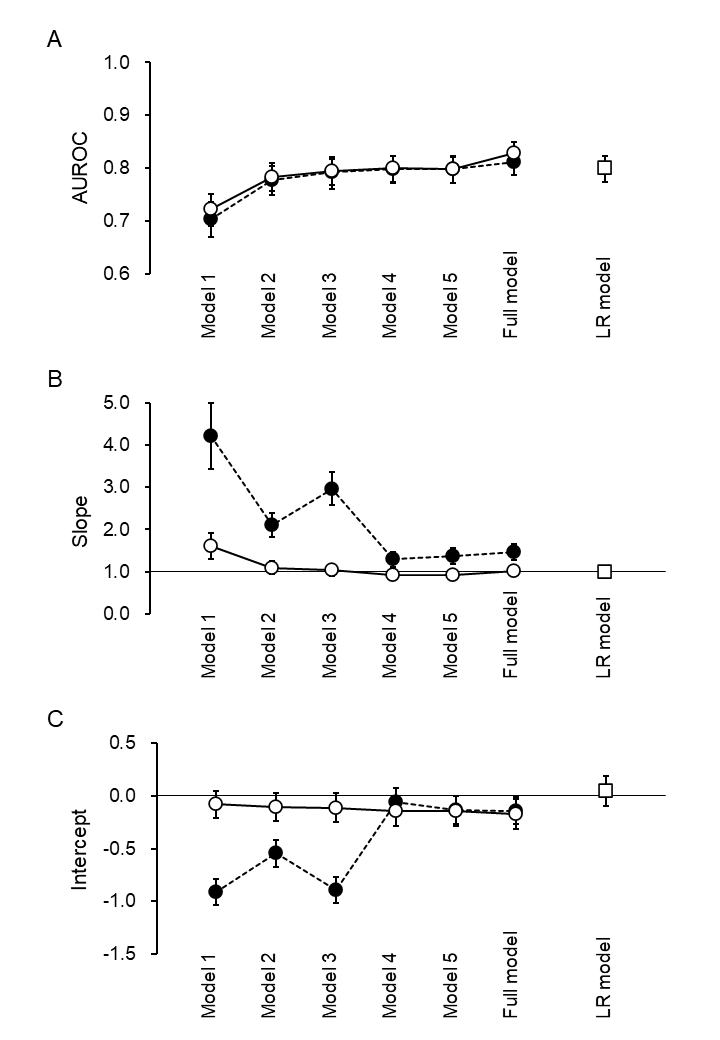


AUROC: area under the receiver operating characteristic curve, LR: logistic regression. The AUROC (A), calibration slope (B), and calibration intercept (C) are shown for an increase in the number of predictors in the XGBoost model (closed circles) and LASSO model (open circles) in comparison with the logistic regression model with pre-specified predictors (open squares). The predictors used in the logistic regression model were selected when their importance was ranked in the top 10 for both the XGBoost and LASSO models. The predictors used in each model are as follows. Model 1: age, Model 2: model 1 + intensive care unit, Model 3: model 2 + Glasgow Coma Scale score, Model 4: model 3 + anesthesia time, Model 5: model 4 + blood loss during surgery, Full model: all 55 variables.

**Figure S2**. Variable importance for delirium after emergent surgery in the machine learning models.


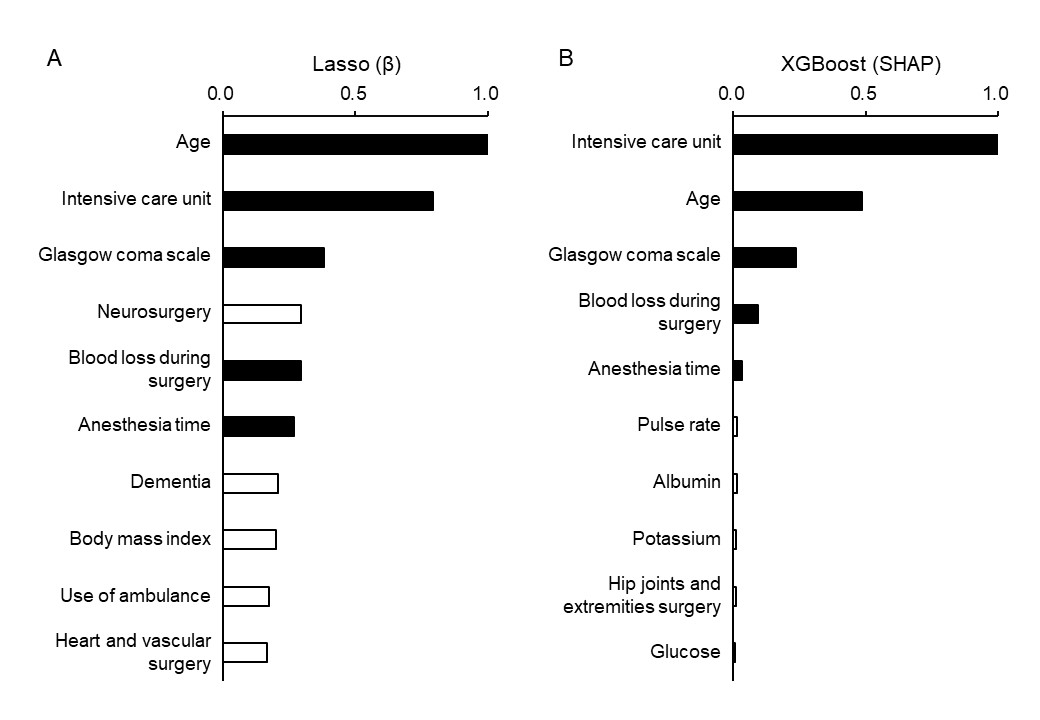


The graph shows the variable importance of the top 10 ranked predictors in the LASSO (A) and XGBoost (B) models. Variable importance was assessed based on the standardized regression coefficient (β) for the LASSO model and the SHAP value for the XGBoost model and is depicted as the value relative to the highest value. Solid bars indicate the variables that are ranked in the top 10 for both models.
